# Trigeminal Neuralgia As the Principal Manifestation of Anaplasmosis: A Case Report

**DOI:** 10.7759/cureus.21668

**Published:** 2022-01-27

**Authors:** Michaela J LeDonne, Sultan A Ahmed, Scott M Keeney, Howard Nadworny

**Affiliations:** 1 Research, Lake Erie College of Osteopathic Medicine, Erie, USA; 2 Internal Medicine, Apogee Physicians, Erie, USA; 3 Internal Medicine, Lake Erie College of Osteopathic Medicine, Erie, USA; 4 Infectious Diseases, Saint Vincent Hospital, Erie, USA

**Keywords:** infectious disease, neurology, case report, tick-borne, anaplasmosis, trigeminal neuralgia

## Abstract

Unlike the tick-borne diseases ehrlichiosis and Lyme disease, human granulocytic anaplasmosis is rarely associated with neurological complications. In this case report, we present a patient who developed a severe, lancinating headache shortly after known tick exposure. A tick-borne PCR panel was positive for *Anaplasmosis phagocytophilum* and neurology evaluation yielded a concomitant diagnosis of new-onset trigeminal neuralgia. Our case explores the relationship between anaplasmosis infection and trigeminal neuralgia.

## Introduction

Human granulocytic anaplasmosis infection, known more simply as anaplasmosis, is a tick-borne disease carried by the *Ixodes scapularis* tick. Epidemiologically, anaplasmosis is more frequently diagnosed in the spring and summer months and is most common in the northeast United States [1]. Because of climate change and the expansion of the *Ixodes* tick in this region, cases of anaplasmosis and other tick-borne diseases are predicted to increase greatly over the coming years [2]. Anaplasmosis has a short incubation period, with symptoms appearing an average of 5.5 days after tick exposure. Common symptoms include fever, chills, malaise, myalgias, and headache, though more severe infection can occur in patients of advanced age, with comorbid conditions, or with compromised immune systems [3]. Neurological symptoms are rare but have been reported with anaplasmosis and include meningoencephalitis, cranial nerve palsies, and demyelinating polyneuropathies [4]. Laboratory findings typical of anaplasmosis infection include leukopenia, thrombocytopenia, and elevated transaminases. CSF studies are typically within normal limits, regardless of neurological symptoms. There are a variety of diagnostic tests for anaplasmosis, including indirect fluorescent antibody testing, serology using ELISA, or DNA amplification through PCR. Treatment should include doxycycline for 7-10 days and should be initiated in all symptomatic patients with suspected disease [3]. The following documents a case of bilateral trigeminal neuralgia triggered by anaplasmosis.

## Case presentation

The patient is an 80-year-old female with a past medical history of hereditary hemochromatosis, hypertension, and gastroesophageal reflux disease who presented to the hospital with a sudden onset of severe, lancinating headache in the distribution of the fifth cranial nerve bilaterally. She also reported intermittent fever, with a maximum temperature of 102°F, fatigue, myalgias, and joint pain. Two months prior, the patient had completed a two-week course of doxycycline for presumed Lyme disease after being bitten by a tick and noticing a rash on her torso. She reported frequent tick exposures at home, as she lives in a wooded area, and stated that in the time after her antibiotic completion, she did find another non-engorged tick that was easy to remove.

On initial evaluation, the patient’s vitals showed a temperature of 97.2°F, blood pressure of 133/74 mmHg, heart rate of 107 beats per minute, respiratory rate of 17 breaths per minute, and an oxygen saturation of 99% on room air. The complete blood count revealed lymphopenia with a white blood cell count of 3.10 k/mcL and thrombocytopenia with a platelet count of 99,000 k/mcL. A basic metabolic panel showed mild hyponatremia with a sodium of 131 mmol/L. A hepatic function panel demonstrated elevated alanine and aspartate aminotransferases of 54 and 62 U/L, respectively. C-reactive protein was elevated at 108.9 mg/L. Both computed tomography of the head without contrast and computed tomography angiography of the neck with contrast were unremarkable. Lumbar puncture was performed with cerebrospinal fluid analysis that was negative for viral and bacterial infections, including Lyme disease. Specifically, CSF showed no RBCs or nucleated cells, glucose was 65 mg/dL, and protein was 20 mg/dL. Neurology consultation was obtained and the patient’s symptoms were felt to be consistent with a diagnosis of trigeminal neuralgia. MRI was recommended, but the patient was unable to undergo MRI because of her history of stapedectomy with steel stapes implant in the 1980s. Upon further questioning, the patient also revealed a family history of trigeminal neuralgia in her mother. 

To further evaluate the cause of her symptoms and laboratory abnormalities, infectious disease was consulted. Given her history of frequent tick exposures, it was felt that anaplasmosis was possible. Given its shorter incubation period and the fact that erythema migrans would likely precede any neurologic symptoms, a diagnosis of Lyme disease was considered less likely when taking into account the patient’s recent completion of a two-week course of doxycycline. However, anaplasmosis and ehrlichiosis can both develop over a shorter timeframe and without a noticeable rash, making these infections a more likely explanation of the patient’s signs and symptoms. To confirm the suspected diagnosis, a tick-borne disease panel was ordered and was positive for *Anaplasma phagocytophilum*  DNA by PCR. *Babesia microti* DNA, *Borriela miyamotoi *DNA, *Ehrlichia chaffeensis* DNA, and *Borrelia burgdorferi* DNA were not detected. 

The patient was treated with 300 mg of gabapentin twice daily and 100 mg of doxycycline twice daily for two weeks. One week following her discharge, laboratory tests were repeated. A complete blood count revealed resolution of her leukopenia and thrombocytopenia. Hepatic function tests showed normalization of aspartate aminotransferase to 39 U/L and improvements of alanine aminotransferase to 40 U/L. CRP was also within normal limits at 1.8 mg/L. She also reported marked improvement of her headache.

## Discussion

**Figure 1:**
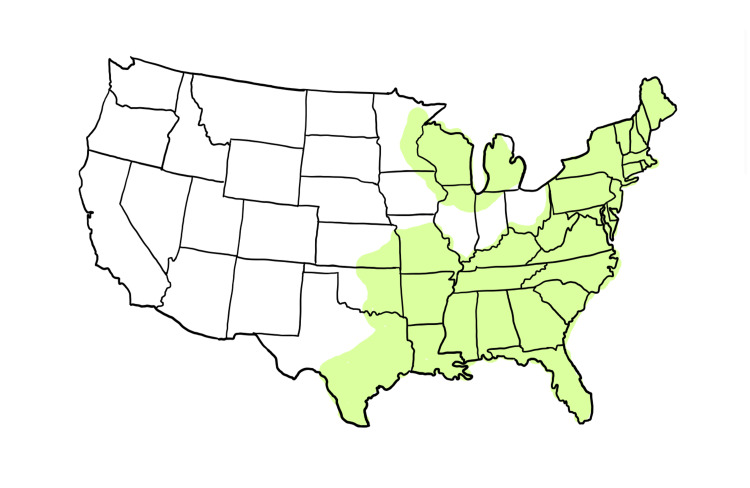
Geographic distribution of the Ixodes Scapularis tick, the main reservoir for anaplasmosis infection.

Anaplasmosis, caused by *Anaplasma phagocytophilum*, is a vector transmitted disease noted in forest regions across the United States. Along the Eastern coast, the most common vector is the *Ixodes Scapularis* tick (Figure 1), which can also co-transmit *Borrelia burgdorferi* (the bacteria responsible for Lyme Disease). A tick needs to be attached for 36-48 hours for Borrelia to be transmitted and 4-24 hours for Anaplasmosis [1,3]. 

Anaplasmosis presents with non-specific signs and symptoms including fever, myalgia, and headache [4]. The symptoms can vary in their presentation and time of recovery from subclinical and self-limited to subacute and prolonged. Some laboratory results suggestive of anaplasmosis include leukopenia, thrombocytopenia, elevated transaminases, and elevated lactate dehydrogenase. Unlike Lyme Disease, Anaplasmosis does not commonly present with a rash. Furthermore, neurological pathology is also rare, and patients that do manifest neurological signs and symptoms will have non-significant findings on cerebrospinal fluid analysis [5]. Rare cases of Anaplasmosis showcase post-infectious complications such as demyelinating polyneuropathy and brachial plexopathy [6]. Although severe headache is a common presenting symptom in patients with anaplasmosis, prior studies have not linked anaplasmosis and trigeminal neuralgia. Our case suggests that anaplasmosis was the cause of our patient’s new-onset trigeminal neuralgia.

Trigeminal neuralgia is a specific type of headache that refers to unilateral, recurrent brief episodes of shock-like pain in the distribution of the trigeminal nerve [7,8]. This can occur in the sub-divisions of cranial nerve V, primarily seen in V2 and V3 but V1 is also possible [9]. Pain can be spontaneous or provoked by either touching trigger zones that match the distribution of these nerves or performing movements such as brushing of teeth or shaving. In some cases, smiling or grimacing can be sufficient to trigger pain [10, 11]. Patients may describe the pain as a sudden, stabbing sensation that occurs repetitively throughout the day, but others might have milder pain that is more continuous [7,8,12]. Less commonly, patients have recorded bilateral sensitivity. In chronic cases, unilaterally presenting symptoms can progress to neuralgia bilaterally [9]. Bilateral trigeminal neuralgia on initial encounter has been seen in 10 percent of cases [13].

**Figure 2:**
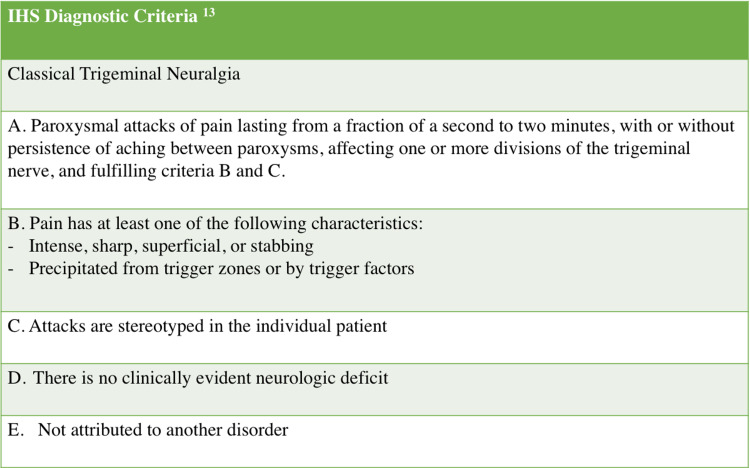
Diagnostic criteria of trigeminal neuralgia.

**Figure 3:**
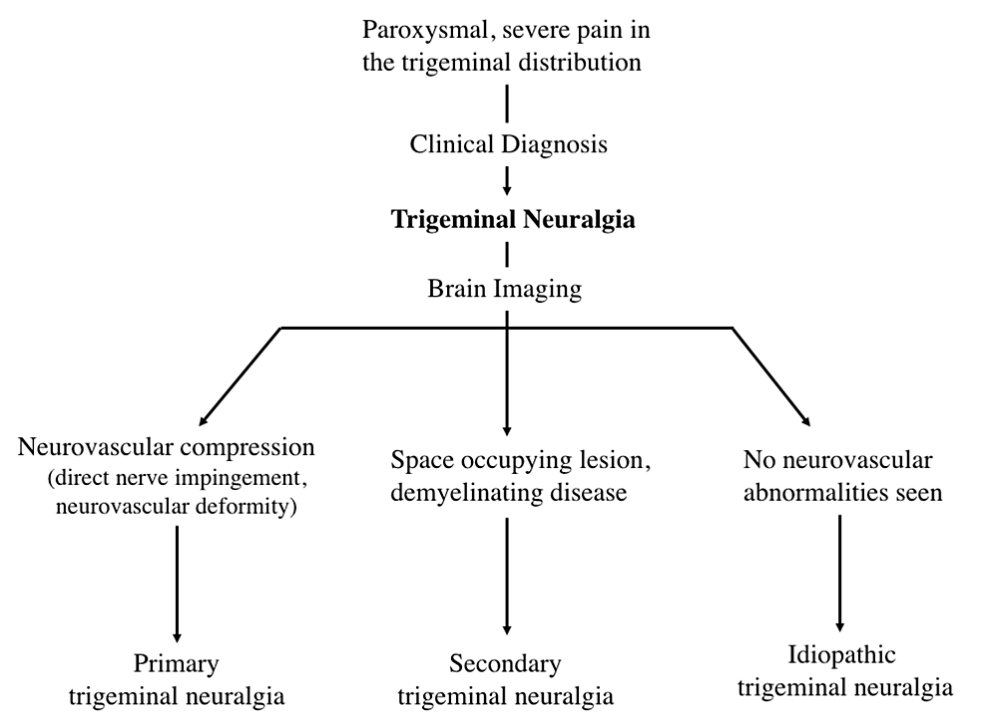
Clinical diagnosis and flow chart that can be used to determine the cause of trigeminal neuralgia.

Trigeminal neuralgia is diagnosed clinically using specific criteria (Figure 2). Brain imaging can then be used to help determine the cause. MRI with and without contrast is the imaging modality of choice to rule out neurovascular compression of the trigeminal nerve, but CT can be used as an alternative [14]. Causes of trigeminal neuralgia include compression of the trigeminal nerve at the root, damage to the nerve secondary to multiple sclerosis, or through demyelination of the trigeminal nerve pathways [15,16,17]. Once these causes are ruled out, many cases are considered idiopathic (Figure 3). Additionally, there is a likely genetic transmission of a predisposition for trigeminal neuralgia via mechanisms such as abnormalities of sodium channels or dysfunction of nociceptors that lead to sensitization [18]. 

Neurological complications of tick-borne diseases are more frequently linked to Lyme disease or ehrlichiosis. Lyme disease can present with symptoms of meningitis such as neck stiffness, headache, and facial palsy [3]. Ehrlichiosis can present with several CNS symptoms such as altered mental status and meningoencephalitis in late illness [3]. Anaplasmosis, however, is only rarely linked to neurological complications such as demyelinating polyneuropathy and brachial plexopathy in late disease. Given our patient’s history of prior Lyme disease, frequent tick exposure, and family history of trigeminal neuralgia, it is interesting to consider how the newly diagnosed anaplasmosis infection triggered the presentation of trigeminal neuralgia. Recent studies have explored the relationship between inflammation and trigeminal neuralgia, specifically showing increased levels of inflammatory markers in those with trigeminal neuralgia compared to healthy controls [19]. In addition, certain pro-inflammatory cytokines, like TNF-a, IL-17, and IL-6, were also found to be elevated in the saliva of trigeminal neuralgia patients compared to controls [20]. Although further research is needed to confirm this connection, it is a possible explanation for trigeminal neuralgia in this patient.

## Conclusions

In a patient presenting to the hospital with non-specific symptoms of fever, myalgia, and headache, clinicians in tick endemic areas should have concern for Lyme disease, anaplasmosis, and ehrlichiosis, regardless of whether the patient is able to recall a known tick bite or pathognomonic rash. Although specific neurological symptoms can vary and have not been consistently linked with various tick-borne illnesses outside of Lyme disease, severe headache is a commonly reported symptom seen in anaplasmosis infection. However, this patient’s presentation of anaplasmosis with new onset trigeminal neuralgia appears to be unique and rare.

## References

[1] (2021). Center for Disease Control and Prevention: Anaplasmosis transmission. https://www.cdc.gov/anaplasmosis/transmission/index.html.

[2] Dumic I, Severnini E (2018). "Ticking bomb": The impact of climate change on the incidence of Lyme disease. Can J Infect Dis Med Microbiol.

[3] (2021). Centers for Disease Control and Prevention: Anaplasmosis signs and symtpoms. https://www.cdc.gov/anaplasmosis/symptoms/index.html.

[4] Guzman N, Yarrarapu SS, Beidas SO (2021). Anaplasma Phagocytophilum. StatPearls.

[5] Bakken JS, Dumler S (2008). Human granulocytic anaplasmosis. Infect Dis Clin North Am.

[6] Horowitz HW, Marks SJ, Weintraub M, Dumler JS (1996). Brachial plexopathy associated with human granulocytic ehrlichiosis. Neurology.

[7] Cruccu G, Di Stefano G, Truini A (2020). Trigeminal neuralgia. N Engl J Med.

[8] Zakrzewska JM, Linskey ME (2014). Trigeminal neuralgia. BMJ.

[9] Maarbjerg S, Gozalov A, Olesen J, Bendtsen L (2014). Concomitant persistent pain in classical trigeminal neuralgia--evidence for different subtypes. Headache.

[10] Maarbjerg S, Gozalov A, Olesen J, Bendtsen L (2014). Trigeminal neuralgia--a prospective systematic study of clinical characteristics in 158 patients. Headache.

[11] Di Stefano G, Maarbjerg S, Nurmikko T, Truini A, Cruccu G (2018). Triggering trigeminal neuralgia. Cephalalgia.

[12] Love S, Coakham HB (2001). Trigeminal neuralgia: pathology and pathogenesis. Brain.

[13] Cruccu G, Finnerup NB, Jensen TS (2016). Trigeminal neuralgia: New classification and diagnostic grading for practice and research. Neurology.

[14] Cruccu G (2017). Trigeminal neuralgia. Continuum (Minneap Minn).

[15] Antonini G, Di Pasquale A, Cruccu G (2014). Magnetic resonance imaging contribution for diagnosing symptomatic neurovascular contact in classical trigeminal neuralgia: a blinded case-control study and meta-analysis. Pain.

[16] Love S, Hilton DA, Coakham HB (1998). Central demyelination of the Vth nerve root in trigeminal neuralgia associated with vascular compression. Brain Pathol.

[17] Hilton DA, Love S, Gradidge T, Coakham HB (1994). Pathological findings associated with trigeminal neuralgia caused by vascular compression. Neurosurgery.

[18] Mannerak MA, Lashkarivand A, Eide PK (2021). Trigeminal neuralgia and genetics: A systematic review. Mol Pain.

[19] Yao Y, Chang B, Li S (2020). Relationship of inflammation with trigeminal neuralgia. J Craniofac Surg.

[20] Patil S, Testarelli L (2021). Assessment of growth factors, cytokines, and cellular markers in saliva of patients with trigeminal neuralgia. Molecules.

